# Distinct cortical networks for hand movement initiation and directional processing: An EEG study

**DOI:** 10.1016/j.neuroimage.2020.117076

**Published:** 2020-10-15

**Authors:** Reinmar J. Kobler, Elizaveta Kolesnichenko, Andreea I. Sburlea, Gernot R. Müller-Putz

**Affiliations:** a Institute of Neural Engineering, Graz University of Technology, Graz, Styria, 8010, Austria; b Swammerdam Institute for Life Sciences–Center for Neuroscience, University of Amsterdam, Amsterdam, North Holland, 1098XH, the Netherlands; c BioTechMed, Graz, 8010, Styria, Austria

**Keywords:** EEG, Movement-related cortical potential, Movement direction, Sensorimotor cortex, Parieto-occipital cortex, Visuomotor task

## Abstract

Movement preparation and initiation have been shown to involve large scale brain networks. Recent findings suggest that movement preparation and initiation are represented in functionally distinct cortical networks. In electroencephalographic (EEG) recordings, movement initiation is reflected as a strong negative potential at medial central channels that is phase-locked to the movement onset - the movement-related cortical potential (MRCP). Movement preparation describes the process of transforming high level movement goals to low level commands. An integral part of this transformation process is directional processing (i.e., where to move). The processing of movement direction during visuomotor and oculomotor tasks is associated with medial parieto-occipital cortex (PO) activity, phase-locked to the presentation of potential movement goals. We surmised that the network generating the MRCP (movement initiation) would encode less information about movement direction than the parieto-occipital network processing movement direction. Here, we studied delta band EEG activity during center-out reaching movements (2D; 4 directions) in visuomotor and oculomotor tasks. In 15 healthy participants, we found a consistent representation of movement direction in PO 300–400 ​ms after the direction cue irrespective of the task. Despite generating the MRCP, sensorimotor areas (SM) encoded less information about the movement direction than PO. Moreover, the encoded directional information in SM was less consistent across participants and specific to the visuomotor task. In a classification approach, we could infer the four movement directions from the delta band EEG activity with moderate accuracies up to 55.9%. The accuracies for cue-aligned data were significantly higher than for movement onset-aligned data in either task, which also suggests a stronger representation of movement direction during movement preparation. Therefore, we present direct evidence that EEG delta band amplitude modulations carry information about both arm movement initiation and movement direction, and that they are represented in two distinct cortical networks.

## Highlights

• Delta band EEG carries information about arm movement initiation and direction.
• Movement initiation and direction are represented in two distinct cortical networks.
• Information about movement direction is primarily encoded in parieto-occipital areas.
• The activity in parieto-occipital areas is phase-locked to the direction cue.

## 1 Introduction

Whenever a reaching movement is made, a series of processes occur in the brain that include cue processing, extraction of high level movement goals, computation of low-level details (Battaglia-Mayer, 2018; Kalaska and Crammond, 1992) and deciding to initiate the movement (Cisek and Kalaska, 2010; Rizzolatti and Luppino, 2001). Recent evidence from behavioral studies with humans and spiking activity in non-human primates suggests that preparation and initiation of movements are reflected by parallel neural processes (Carland et al., 2019; Haith et al., 2016). Movement preparation involves a series of processes related to cue perception, determination of high-level movement goals (e.g., where to move), and their translation to low-level movement commands, whereas movement initiation reflects decision processes dealing with the commitment to move to a particular movement goal (e.g., when to move) and the vigor of the ensuing movement (Carland et al., 2019; Haith et al., 2016). Movement initiation is typically accompanied with strong activity in sensorimotor areas and basal ganglia phase-locked to the movement onset (Carland et al., 2019; Kaufman et al., 2016; Pfurtscheller and Lopes Da Silva, 1999; Shibasaki et al., 1980), while movement preparation is associated with fronto-parietal network activity, transforming high-level movement goals to low-level commands (Cavina-Pratesi et al., 2018; Cisek and Kalaska, 2010; Fabbri et al., 2010; Ledberg et al., 2007; Perry and Fallah, 2017). It is established that spiking activity in primary sensorimotor areas carries information about movement direction at the movement onset (Georgopoulos et al., 1982). It is not clear whether macroscopic activity in terms of electroencephalographic (EEG) signals carry directional information phase-locked to the movement onset.

Within the EEG activity, time-domain amplitude modulations in the delta band that start prior to the movement onset have been reported to be predictive for movement initiation (Bierbaumer et al., 1990, Deecke et al., 1969, Shibasaki and Hallet, 2006). In the context of a motor task, slow potentials in the EEG activity phase-locked to the movement onset are known as movement-related cortical potentials (MRCPs) (Deecke et al., 1969). MRCPs have been investigated in relation to lower-limb movements, such as isometric dorsiflexion (Xu et al., 2014) or walking (do Nascimento et al., 2005; Jiang et al., 2015; Sburlea et al., 2015), as well as in upper-limb movements (Ibáñez et al., 2014; López-Larraz et al., 2014; Niazi et al., 2011). Several studies involving different types of movement have shown that amplitude and phase information encoded in the time-domain delta band are predictive of movement initiation (Sburlea et al., 2017; Sun et al., 2017).

Apart from encoding the initiation of executed (Lew et al., 2012), attempted (Ofner et al., 2019) and imagined (Pereira et al., 2018) upper-limb movements, MRCPs have also been reported to encode information about goal-directedness (Pereira et al., 2017), speed (Gu et al., 2009), force (Jochumsen et al., 2013), grasp types (Schwarz et al., 2018), other movements of the upper limb (Ofner et al., 2017, Ofner et al., 2019) and movement direction (Li et al., 2012, Waldert et al., 2008). The representation of directional information within the MRCP is typically studied during center-out reaching tasks and aligned to the response (Robinson and Vinod, 2016). The employed experimental paradigms often force the participants to respond after a go cue which leads to considerable phase locking between the direction cue and the response (Waldert et al., 2008). Moreover, higher classification accuracies for movement direction can be obtained if the participants are allowed to move immediately after target presentation rather than a forced random delay period (Wang et al., 2010).

Since movement initiation and preparation are likely to be independent processes in the brain (Haith et al., 2016), it is not clear if the time-domain amplitude modulations encode information about movement direction phase-locked to the movement onset (movement initiation) or to the cue (movement preparation). Knowing that directional processing is an integral part of movement preparation, we surmise that delta band EEG activity has a stronger representation of movement direction during movement preparation (i.e., phase-locked to the cue) than during movement initiation (i.e., phase-locked to the movement onset). If this is the case, a classifier should predict the movement direction with higher accuracy in the cue aligned case compared to the movement onset aligned case. To test our hypothesis, we conducted an EEG study with healthy participants. Our experimental paradigm comprised a center-out task to identify cortical regions encoding directional information, and two experimental conditions to identify cortical regions generating the MRCP associated with arm movements. In the first condition (observation), the participants moved their eyes; in the second condition (execution), they also moved their right arm.

## 2 Materials and methods

### 2.1 Participants

Fifteen healthy participants were recruited for this study (23.8 (mean) ​± ​2.8 (standard deviation, SD) years, 9 female). Eleven participants had already participated at least once in an EEG experiment before. All participants had normal or corrected to normal vision, and self-reported to be right handed. The study was carried out in accordance with the Declaration of Helsinki and was approved by the ethics committee of the Medical University of Graz. All participants were instructed about the purpose and procedure of the study, after which they signed an informed consent form. All received monetary compensation for their participation.

### 2.2 Experimental set-up

**Fig. 1:**
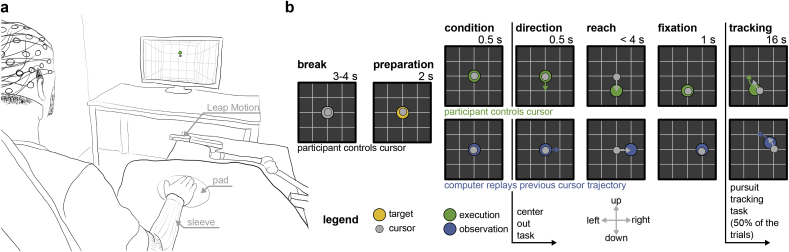
Experimental setup and paradigm. **a,** During the experiment, the participants sat in a comfortable chair in front of a computer screen. Both arms were supported at the same height. Right arm movements were recorded with a LeapMotion controller. Forward/backward hand movements on the table were mapped to upward/downward cursor movements on the screen. **b,** Diagram of the experimental paradigm. A visual cue indicated the condition, either execution (green target) or observation (blue target), followed by a 2D center-out task in four directions. The direction was indicated by the target movement (0.5 ​s duration; arrows visualize movement in the individual images). After 1 ​s of fixation, a pursuit tracking task was performed for 16 ​s (50% of the trials). In the execution condition the participants controlled the cursor, while in the observation condition, the computer replayed a previously executed cursor trajectory that matched the current target trajectory.

During the course of the experiment, the participants were sitting in a comfortable chair, positioned 1.4 ​m away from a computer screen (Fig. 1a). An armrest supported the left arm, while the right arm was supported by a table at the same height. To reduce friction between the right arm and the table, participants were asked to wear a sleeve and place their hand on a circular pad. A LeapMotion controller (LeapMotion Inc., San Francisco, USA), placed 20 ​cm above the hand, was used to record the right hand’s palm position. After each participant found a comfortable resting position, the palm position was mapped to the origin of a virtual environment (center of the screen). In analogy to the interaction with a computer by using a computer mouse, we decided to map rightward/forward hand movements to rightward/upward cursor movements. We mapped a circle with a 5-cm radius around the resting position to a circle with a 16-cm radius on the screen. The limits of the circle on the screen were indicated by the bounds of a virtual grid. For instance, a right hand movement 5 ​cm to the right would make the cursor touch the grid on the right side.

### 2.3 Experimental procedure

The experimental procedure consisted of 4 blocks, and lasted 3 ​h in total. In the first block, we asked the participants to familiarize themselves with the paradigm (approx. 10 min). In the second and fourth blocks, eye artifacts (blinks and eye movements) and resting activity were recorded (approx. 5 min each). The detailed procedure is described in Kobler et al. (2017). In the third block, participants performed the main experimental task according to the paradigm illustrated in Fig. 1b. Each trial implemented a two dimensional (2D), visually guided center-out reaching task (COT). In half of the trials, the COT was followed by a pursuit tracking task (PTT), which we used to study the tuning characteristics of low-frequency EEG signals to end-effector positions and velocities (Kobler et al., 2018).

A trial started if a target stimulus (large sphere) changed its color to yellow. This triggered the participants to focus their gaze upon the target and to keep their hand in the resting position and, thereby, the cursor (small gray sphere) in the center of the screen. Our paradigm distinguished between two conditions: observation (obs) and execution (exe). The conditions were indicated by a visual cue (change in target color; pseudo-randomly distributed across trials). After a period of 0.5 ​s, the target started to move in one of four directions (up, right, down, left; pseudo-randomly distributed across trials). After the target moved for 0.5 ​s, it stopped at a distance of 5 ​cm and waited for the approaching cursor. As soon as the distance to the cursor was smaller than a threshold, the fixation period (1 ​s duration) started. In the execution condition (green target), the participants tracked the target visually and controlled the cursor by moving their right hand (visuomotor task). In the observation condition (blue target), the participants tracked the target only visually while their right hand was resting (oculomotor task). We replayed previous, matching cursor trajectories to obtain similar visual dynamics during both conditions. The replay procedure was implemented in an adaptive fashion that ensured similar cursor movements within a participant, and that the participants could neither predict the condition nor the direction of the next trial. The detailed procedure is described in (Kobler et al., 2018).

We asked the participants to make a single smooth, continuous reaching movement towards the target and stop at the target position during the execution condition. If a participant did not move for 4 ​s after the direction cue the trial was aborted. A participant did not move, if the distance between the cursor and the origin was smaller than a threshold (1 ​cm). A trial was also aborted, if the threshold was exceeded while the target was still moving, or during the entire COT in the observation condition. Aborted trials were appended to a queue of trials. A total of 360 trials (180 per condition, pseudo randomly distributed) were recorded in 20 runs with short breaks in between. We recorded only 192 trials for participant 1.

### 2.4 EEG recording

We placed 64 active electrodes (actiCAP, Brain Products GmbH, Gilching, Germany) on the scalp according to the 10–10 system. The reference and ground electrodes were positioned at the right mastoid and AFz, respectively. Six additional active electrodes were placed at the superior, inferior and outer canthi of the right and left eyes to record electrooculographic (EOG) activity. Supplementary Fig. 1 visualizes the locations of all 70 electrodes. After the electrodes were placed on the scalp, their 3D positions and the positions of anatomical landmarks (nasion, left-/right preauricular points) were recorded with an ultrasound based digitizer (ELPOS, Zebris Medical GmbH, Isny, Germany). EEG and EOG signals were recorded at 1 ​kHz (BrainAmp, Brain Products GmbH, Gilching, Germany). The paradigm was implemented in Python 2.7 based on the simulation and neuroscience application (SNAP) platform (Kothe, 2012) and the 3D engine Panda3D (Goslin and Mine, 2004). The screen position signals of the visual stimuli (cursor, target) were recorded at 60 ​Hz. All signals (EEG, EOG and stimuli) were recorded using the lab streaming layer (LSL) protocol (Kothe et al., 2019) and synchronized offline using a photodiode signal, which captured an impulse on the screen at the start of each trial. All signals were then resampled to 200 ​Hz.

### 2.5 Pre-processing

**Fig. 2:**
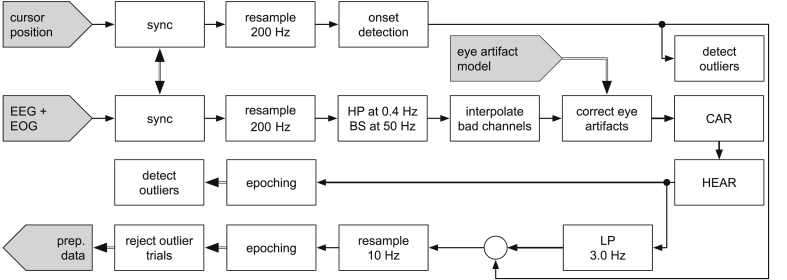
Pre-processing pipeline. After synchronization, EEG and EOG signals were resampled, high-pass and notch filtered; bad channels were spherically interpolated. Next, eye artifacts were corrected. The corrected EEG signals were re-referenced to the common average reference. Then, HEAR was applied to remove transient, single electrode outliers. A subsequent low-pass filter was applied to extract delta band EEG signals. After synchronization, the movement onset was detected using the Euclidean distance between the cursor and the origin. Outlier trials were automatically detected based on the movement onsets and the epoched broadband EEG signals. The final pre-processed dataset comprised the delta band EEG signals during non-outlier trials.

The recorded data was analyzed offline using Matlab (Matlab 2015b, Mathworks Inc., Natick, USA) and the open source software EEGLAB (Delorme and Makeig, 2004) version 14.1.1. Fig. 2 outlines the pre-processing pipeline. The EEG signals were high-pass filtered (0.4 ​Hz cut-off frequency, Butterworth filter, fourth order, zero-phase). Data cleaning was initiated by a spherical interpolation of channels with poor signal quality (visual inspection). We interpolated 2.1 channels on average (Supplementary Table 1). Eye movements and blinks were attenuated by applying the sparse generalized eye artifact subspace subtraction algorithm (Kobler et al., 2020b). The algorithm was fitted to calibration data, recorded during the experimental blocks 2 and 4. The EEG channels were subsequently converted to a common average reference (CAR). We applied the high-variance electrode artifact removal (HEAR) algorithm to attenuate occasional, single electrode pops and low-frequency drifts (Kobler et al., 2019). HEAR monitors the variance of each EEG channel. If the variance increases drastically (e.g., 3 times the variance of calibration data), the probability of an artifact contaminating the signal rises. This artifact probability is then used to weigh the amount of linear interpolation by neighboring channels. Next, a low-pass filter (3.0 ​Hz cut-off frequency, Butterworth filter, fourth order, zero-phase) was applied to the broadband EEG signals to extract delta band activity, before the signals were resampled at 10 ​Hz.

The cursor position signal was used to detect the onset of the reaching movements. For each trial, the cursor movement onset was calculated based on the Euclidean distance between the cursor and the origin. A distance equal to 0 corresponded to the origin (i.e., center position), while a distance equal to 1 indicated the position of the circle bounded by the virtual grid (Fig. 1). During the preparation period the distance was not exactly zero because the resting position was controlled by the participant (Fig. 1b). To remove these trial-to-trial fluctuations, we subtracted the average distance during the last second of the preparation period. The first time-point at which the cursor distance exceeded a threshold of 0.05 served as an initial estimate for the movement onset. It was refined using a window that captured the smoothed (moving average finite impulse response filter, triangular window, 63 filter taps, zero-phase) cursor distance of the preceding 1.75 ​s. For each time-point within the window, the derivative was estimated by computing finite differences. For the time-point with the maximal derivative, we computed the tangent. We defined the time-point at which the tangent intersected with the zero-line along the time axis as the final estimate of the cursor movement onset.

We set the cursor movement offset to the time-point at which the distance between the cursor and final target position became smaller than 0.1 (corresponding to 1.6 ​cm on the screen). The movement duration was then the difference between the estimated offset and onset. Trials with abnormal movement onset or duration were marked as outliers. Abnormal trials were detected if the cursor movement onset relative to the start of a trial (i.e., target turned yellow) or the movement duration were improbable (i.e., larger than 2.5 standard deviations around the mean). The underlying distribution’s mean and standard deviation were computed using robust estimates. In detail, the mean and standard deviation were estimated as the median and 1.4826 times the median absolute deviation among all trials (Leys et al., 2013).

To detect outliers within the EEG signals, we epoched the broadband (0.4–100 ​Hz) EEG signals according to two alignment cases: start of trial aligned and cursor movement onset aligned. In the start of trial aligned case, we extracted epochs in the [0.75, 4.75] s interval after the start of the trial. Whereas in the cursor movement onset aligned case, we extracted epochs in the [-2, 1.25] s interval around the cursor movement onset. In either case, epochs were marked as outliers if the EEG signal of any channel exceeded a threshold of ±200 ​μV or had an abnormal probability, kurtosis or variance (i.e., larger than x standard deviations around the mean across trials, where x equals 6 in the case of probability or kurtosis, and 5 in the case of variance). We applied the joint probability, kurtosis and variance rejection criteria twice to detect gross outliers in the first iteration, and subtle outliers in the second iteration. Finally, the union of detected outlier epochs was rejected from the low-pass filtered and epoched EEG activity.

### 2.6 Behavioral analysis

In order to understand behavioural dynamics of the participants and eliminate differences of visual stimuli dynamics across conditions or directions as confounds, we analyzed the cursor movement onsets and movement durations. To identify group level effects of condition (execution, observation) and direction (right, left, up, down) in the cursor movement onset time-points, a two-way repeated measures analysis of variance (ANOVA) was performed. The same two-way repeated measures ANOVA was also performed for the cursor movement duration. For both ANOVAs, we computed Mauchly’s tests to identify if the sphericity assumption was violated; q-q-plots were used to inspect if the cursor movement onset and duration followed a Gaussian distribution.

In addition to the reach dynamics, we calculated the onsets of the eye movements. Eye movements typically start with a catch up saccade, as a target stimulus starts moving. To identify the catch up saccade timing, vertical and horizontal EOG derivatives were calculated from the broadband EEG data; the difference between right and left outer canthi was computed for the horizontal EOG derivative, and the difference between left/right superior and inferior electrodes was computed for the vertical EOG left/right derivative. We then computed the average across trials with the same direction. The threshold for the direction specific eye movement onset was the time-point at which the EOG derivative, associated with the direction, exceeded the trial baseline activity (1–1.5 ​s) by 3 standard deviations.

### 2.7 Encoding of condition and direction

To identify EEG activity that encodes information about condition and direction, we fitted a general linear model (GLM) to the recordings of each participant. The GLM contained the factors condition, direction and intercept. We split the direction information into two orthogonal factors (horizontal, vertical), equalized the number of trials per condition and direction, and finally z-scored the factors. The factors were identical for all time-points within a trial, and could be expressed as a $4\;\times n_{trials}$ matrix S

(1) $$S=\left[s_{cond};s_{dir,horz};s_{dir,vert};1\right]$$

with $1\;\times n_{trials}$ vectors $s_{cond}$, $s_{dir,horz}$ and $s_{dir,vert}$ coding the factors and a constant intercept term. For each time-point i, the GLM can be defined as

(2) $$X^{\left(i\right)}=A^{\left(i\right)}\;S\;+E^{\left(i\right)}$$

with an $n_{channels}\;\times n_{trials}$ matrix $X^{\left(i\right)}$ reflecting the EEG activity, an $n_{channels}\;\times 4$ matrix $A^{\left(i\right)}$ reflecting the regression coefficients and an $n_{channels}\;\times n_{trials}$ matrix $E^{\left(i\right)}$ containing the residuals which are assumed to follow a Gaussian distribution. The regression coefficients for the intercept term correspond to the average across all trials. The least squares estimate of $A^{\left(i\right)}$ is

(3) $${\hat{A}}^{\left(i\right)}=C_{XS}^{\left(i\right)}\cdot C_{SS}^{-1}$$

with an $n_{channels}\;\times 4$ cross-covariance matrix $C_{XS}^{\left(i\right)}$ between the pre-processed EEG activity and the predictors, and a 4×4 covariance matrix $C_{\mathrm{SS}}$ between the factors. We used analytical shrinkage regularization to estimate $C_{\mathrm{SS}}$(Schäfer and Strimmer, 2005). The predicted EEG activity is then

(4) $${\hat{X}}^{\left(i\right)}={\hat{A}}^{\left(i\right)}\;S$$

Given (2) and (4), the residuals $E^{\left(i\right)}$ are

(5) $$E^{\left(i\right)}=X^{\left(i\right)}\;-\;{\hat{X}}^{\left(i\right)}$$

We used them to inspect if the GLM assumptions were met. In detail, we plotted the residuals of each channel against the predicted feature activity ${\hat{X}}^{\left(i\right)}$ to check whether the homoscedasticity and linearity assumptions of the GLM were met; q-q-plots were used to check if the residuals followed a Gaussian distribution.

### 2.8 EEG source imaging

We applied EEG source imaging (Michel et al., 2004) to map the EEG signals from the channels to the cortical surface and, thereby, ease neurophysiological interpretation. We used the ICBM152 template boundary element (BEM) head model (Fonov et al., 2011). The BEM comprised three layers (cortex, skull, scalp) with relative conductivities (1, 0.008, 1). The cortex layer was modeled with 5011 voxels. We co-registered the BEM and the digitized EEG electrode positions (Supplementary Fig. 1b) using the three anatomical landmarks (nasion, left and right preauricular points). To compensate deviations between participant and template anatomy, we finalized the co-registration by projecting floating electrodes to the scalp layer. OpenMEEG (Gramfort et al., 2010) was used to compute the forward model for 15033 sources (3 sources per voxel) on the cortical surface. sLORETA (Pascual-Marqui, 2002) was used to estimate the inverse solution. To reduce the effect of sensor noise in the inverse solution, 3 ​min of resting data (similar preprocessing as the reaching data; recorded during experimental blocks 2 and 4) were used to estimate a sensor noise covariance matrix (shrinkage regularization with 10% of the average eigenvalue).

After we projected the subject specific EEG signals to the cortical surface, we normalized them by the global field power (GFP) (Kobler et al., 2020a). We estimated the GFP by randomly selecting a sample from each trial, averaging these samples, and computing the standard deviation across sources. This procedure was repeated 10,000 times. The final subject-specific GFP estimate was set to the median of the 10,000 repetitions. After GFP normalization, we computed group level averages of the activity and regression coefficients on the cortical surface.

We tested the regression coefficients for significant group level activation using permutation tests (Maris and Oostenveld, 2007; Nichols and Holmes, 2002). We used 10,000 permutations to obtain a random distribution. In each permutation, we randomly sign flipped the regression coefficients of the participants before the group averages of the 15,033 sources were computed and the norm for each of the 5011 voxels was extracted. A voxel’s p value was then set to the percentage of random voxel norms that were larger than the observed voxel norm.

To reduce the number of tests and obtain a single representation for the direction factor, we added the voxel norms of the regression coefficients associated with the horizontal and vertical factors. This allowed us to identify voxels that code information about the vertical and/or horizontal factor. For the tests we considered the time-points within the interval spanned by the cue associated with each factor (i.e., condition cue for the condition factor) and the cursor movement offset. The number of tests was 461,012 ​= ​3 (factors) ∗ 2 (alignments) ∗ 5011 (voxels) ∗ 15.3 (average number of time-points).

We also investigated differences in the encoding of direction between the conditions in 8 regions of interest (ROIs) along the dorsal reaching system (Gallivan and Culham, 2015). The ROIs are displayed in Fig. 5a; they covered parieto-occipital cortex (PO), superior parietal lobule (SPL), premotor cortex (PM) and primary sensorimotor cortex (SM1) of both hemispheres. Before the differences were computed and averaged across participants, each voxel’s norm was extracted. In doing so, we identified voxels which encoded more directional information in one condition, irrespective of the participants’ dipole orientations. We used two-sided, permutation (10,000), paired t-tests to identify significant differences. The number of tests was 16 ​= ​8 (ROIs) ∗ 2 (alignments). We controlled the false discovery rate (FDR) at a significance level of 0.05 for a total of 461,028 tests (Benjamini and Yekutieli, 2001).

### 2.9 Classification of condition and direction

We used the pre-processed EEG signals to classify conditions (execution vs. observation) using two-class shrinkage regularized linear discriminant analysis (sLDA) (Blankertz et al., 2011). sLDA is an optimal classifier in the sense that it minimizes the classification error, if the features follow a Gaussian distribution and have the same covariance matrix for all classes. Blankertz et al. showed that these two assumptions typically hold for event-related potential data (Blankertz et al., 2011). The pre-processed EEG signals were split into train and test sets using a leave-one-trial-out cross-validation (CV) scheme. In this scheme, if N is the number of trials, the classifier was trained on N-1 trials, and tested on the held-out trial. The process was repeated over the N folds, so that each trial was tested once.

We fitted and evaluated a classifier for each time-point using two approaches: single time-point and windowed. In the single time-point approach, we classified the activity of the 64 EEG channels, while in the windowed approach we classified the activity of the 64 EEG channels at the current and 5 preceding time-points (64 channels x 6 time-points ​= ​384 features). We computed classification accuracies to summarize the performance on the held-out test sets.

For a balanced, two-class problem with an infinite amount of trials, the theoretical chance level constitutes 50%. Due to the limited number of trials, we estimated the significance level through a shuffling approach. In 1000 repetitions, we randomly permuted class labels across trials and applied the same leave-one-trial-out CV scheme. We set the significance level to the 95 percentile of the test set classification accuracies.

We further sought to determine if directional information can be decoded from low frequency time domain EEG signals. For this purpose, we fitted a 4-class sLDA classifier to discriminate between directions (up, right, down, left). We applied the same leave-one-trial-out CV scheme as for the condition classifier. To identify interaction effects, we repeated the direction classification for each condition.

We used permutation tests to identify significant group level effects (Maris and Oostenveld, 2007; Nichols and Holmes, 2002). As before, we tested time-points within the interval spanned by the cue associated with each factor (i.e., condition cue for the condition factor) and the cursor movement offset. We first tested if the observed classification accuracies were significantly different from chance, by computing one-sided, permutation (10,000 permutations), paired t-tests between the observed accuracies and the participant specific significance threshold (95 percentile of the shuffling results). The number of tests was 106 ​= ​4 (condition, direction, direction (exe), direction (obs)) ∗ 2 (alignment) ∗ 13.3 (average number of time-points). Next, we used two-sided, permutation (10,000 permutations), paired t-tests to test if the direction classification accuracy was significantly different between the conditions. The number of tests was 24 ​= ​2 (alignment) ∗ 12 (time-points). Finally, we computed two-sided, permutation (10,000 permutations), paired t-tests to identify differences in the peak classification accuracy between the two types of alignments (start of the trial, cursor movement onset) in both conditions (2 tests). The total number of tests was 132. We controlled the false discovery rate at a significance level of 0.05 (Benjamini and Yekutieli, 2001).

## 3 Results

### 3.1 Behavioral results

**Fig. 3:**
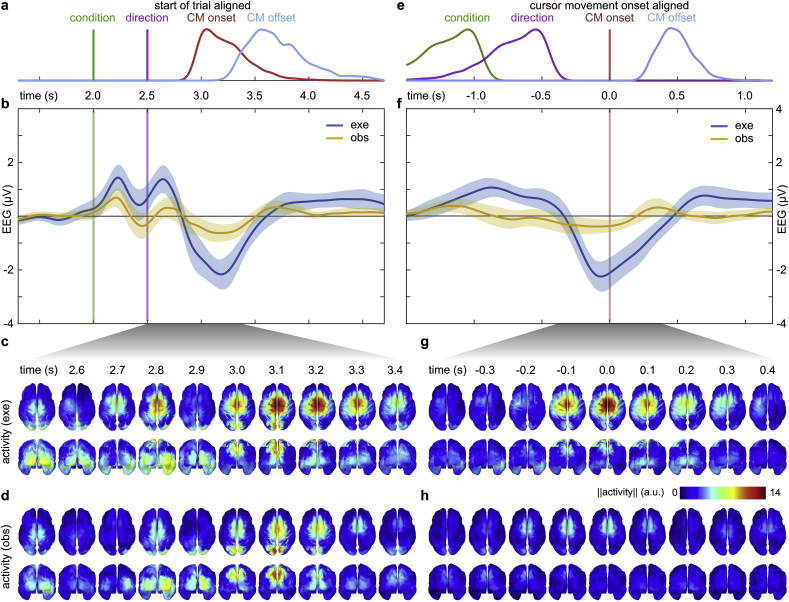
Condition specific grand average activity in channel and source space for start of trial aligned (left) and cursor movement aligned (right) data. **a,** Distribution of the cues and cursor movement (CM) onsets/offsets for start of trial aligned data (t ​= ​0 ​s). A Gaussian smoothing kernel (0.04 ​s bandwidth) was used to estimate the probability density from all trials and participants. The participant level distributions are summarized in Supplementary Fig. 2. **b,** Grand average MRCP for start of trial aligned data at channel C1. The conditions are color-coded. Shaded areas summarize the confidence interval of the mean across participants. **c,** Grand average source space activity for execution condition and time-points around the average cursor movement onset (t ​= ​3.2 ​s). **d,** As in **c** for observation condition. **e-h,** As in **a-d** for cursor movement onset aligned data (t ​= ​0 ​s).

The distribution of cursor movement onsets and offsets are presented in Fig. 3a,e and Supplementary Figs. 2 and 3. The grand average cursor movement onset with respect to the start of a trial was 3.23 ​s (0.18 ​s SD) and the mean cursor movement duration with respect to cursor movement onset was 0.49 ​s (0.13 ​s SD). The cursor movement onset and duration were approximately normally distributed across participants. For the cursor movement onset, Mauchly’s test indicated that the assumption of sphericity had neither been violated for direction (W ​= ​0.73, p ​> ​0.05) nor the interaction of direction and condition (W ​= ​0.87, p ​> ​0.05). For the cursor movement duration, the assumption of sphericity had neither been violated for direction (W ​= ​0.52, p ​> ​0.05) nor the interaction of direction and condition (W ​= ​0.71, p ​> ​0.05). The two-way repeated measures ANOVA showed no significant main effect of condition on the cursor movement onset (F ​= ​3.43, p ​= ​0.085, df ​= ​1) and on the cursor movement duration (F ​= ​1.29, p ​= ​0.27, df ​= ​1). There was also no significant main effect of direction on the cursor movement onset (F ​= ​1.58, p ​= ​0.208, df ​= ​3). We identified a significant main effect of direction on the cursor movement duration (F ​= ​4.99, p ​= ​0.047, df ​= ​3). There was no significant interaction effect between condition and direction on the cursor movement onset (F ​= ​1.09, p ​= ​0.365, df ​= ​3) and on the cursor movement duration (F ​= ​0.56, p ​= ​0.643, df ​= ​3).

To further investigate the main effect of direction on the cursor movement duration, post-hoc pairwise t-tests with Bonferroni correction were performed. The mean cursor movement durations were 0.48 ​s (0.09 ​s SD) for the right direction, 0.51 ​s (0.10 ​s SD) for the left direction, 0.48 ​s (0.08 ​s SD) for the up direction and 0.49 ​s (0.09 ​s SD) for the down direction. The post-hoc tests revealed a significant difference in movement duration between the right and left directions (p ​= ​0.0053), with a difference in means of 0.03 ​s. Despite the significant difference, the effect size remains small. We concluded that condition had no effect on the cursor dynamics, and direction had a negligible effect on the cursor movement duration.

Regarding eye movement behavior, the average time for a catch-up saccade to take place was 2.69 ​s (0.06 ​s SD). Since the target started to move 2.5 ​s after the start of a trial, it means that it took on average 190 ​ms for the eyes to catch up with the moving target. This result is in line with the typical timing of catch-up saccades (Purves et al., 2004).

### 3.2 Movement-related cortical potentials

To compare time-domain amplitude modulations in execution and observation conditions, we computed grand average MRCPs for channel C1 for start of trial aligned data and cursor movement onset aligned data (Fig. 3b,f). In addition to the MRCP, we also compared the source space activity in either condition for selected time points around the cursor movement onset (Fig. 3c,d,g,h).

Looking at the results for the start of trial alignment (left panel in Fig. 3), the activity before the condition cue at 2.0 ​s is fluctuating around 0 ​μV (Fig. 3b). Following the condition cue at 2.0 ​s and the direction cue at 2.5 ​s, there are pronounced visually evoked potentials (VEPs) originating in occipital areas (Fig. 3c and d). After the second VEP, we observed an MRCP in execution condition, with a steep negative amplitude deflection (Fig. 3b) peaking in negativity at the average cursor movement onset (−2.3 ​μV ​at 3.2 ​s). There was also a smaller but noticeable negative amplitude deflection in the observation condition (−0.7 ​μV ​at 3.2 ​s). As expected, the execution condition-specific MRCP led to a stronger activation in contralateral and central motor areas (Fig. 3c) in comparison to the observation condition (Fig. 3d).

For the cursor movement onset alignment (right panel in Fig. 3), the cue-locked VEP effects faded at channel C1 (Fig. 3f). Within the interval −1.0 ​s to −0.5 ​s, we observed a positivity in the execution condition. Starting at −0.4 ​s, there was a prominent MRCP in the execution condition. At channel C1, the MRCP amplitude peaked with a value of −2.3 ​μV ​at −0.1 ​s, corresponding to 100 ​ms before the cursor movement onset (Fig. 3f). The negativity was generated in contralateral and central motor areas (Fig. 3g). In the observation condition, the activation in central motor areas was considerably lower (Fig. 3h). Generally, and as expected, the MRCP related to the hand movement initiation (execution condition) was phase-locked to the cursor movement onset.

### 3.3 Cortical sources encoding condition and direction

**Fig. 4:**
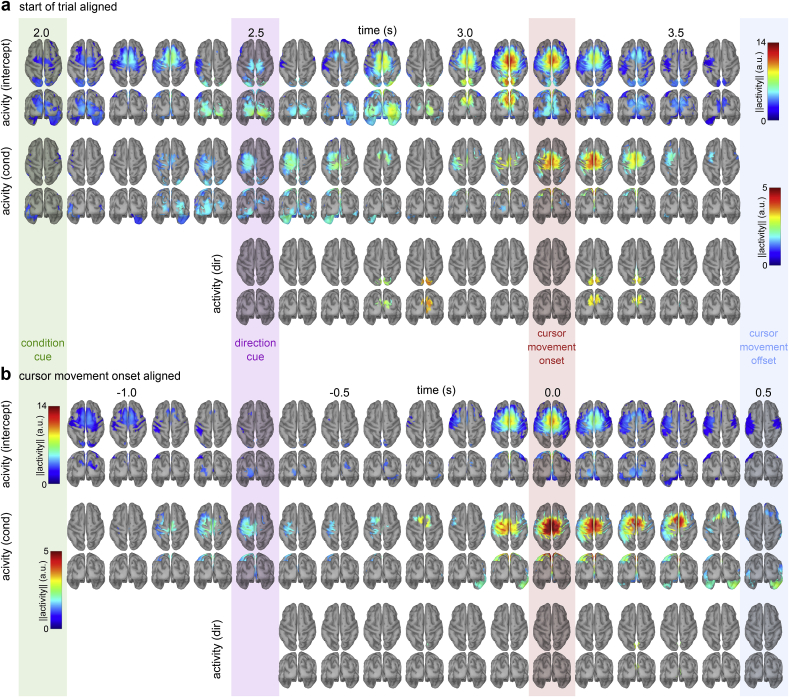
Grand average source space condition and direction factor encoding results for start of trial aligned (**a**) and cursor movement onset aligned (**b**) data. Each plot states the regression coefficients of the linear encoding model at the voxel level. I.e. the activity that is explained by each factor (intercept, condition, direction). The rows summarize the factors and the columns the time-points. Only voxels with significant group level regression coefficients are shown (one-sided permutation tests, critical p-value ​= ​0.0004, FDR corrected).

We used a GLM and EEG source imaging to identify cortical sources encoding information about the condition and direction. The residuals of the GLM generally followed a normal distribution and indicated that the homoscedasticity and linearity assumptions were fulfilled. Fig. 4 displays voxels with significant regression coefficients for the factors intercept, condition and direction.

**Fig. 5:**
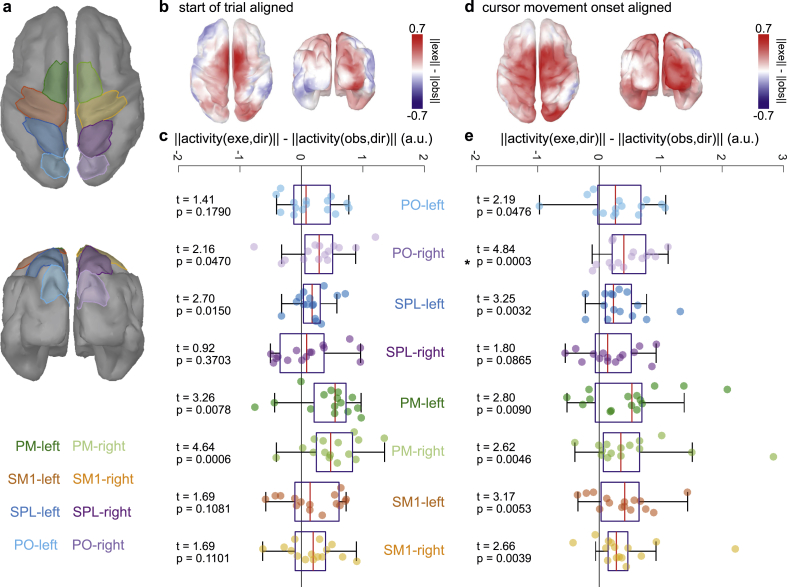
Condition-specific direction encoding around the cursor movement onset. **a,** Regions of interest (ROIs). The ROIs covered parieto-occipital cortex (PO), superior parietal lobule (SPL), primary sensorimotor cortex (SM1) and premotor cortex (PM) of both hemispheres. **b,** Start of trial alignment. Grand average difference in direction-related activity between execution and observation condition. The average within the interval t ​= ​[2.9, 3.4] s is displayed. Before the differences were computed and averaged across participants and time-points, each voxel’s norm was extracted. As a result, voxels which encoded more directional information in one condition, irrespective of the participants’ dipole orientations were identified. Red voxels indicate stronger direction related activity in the execution condition. **c,** Boxplots summarizing **b** at ROI level. Each boxplot displays the difference in direction related activity. The ROIs are color coded. Each dot marks the result of one participant. Two-sided, permutation, paired t-tests did not reveal significant differences (critical p-value ​= ​0.0004, FDR corrected). **d-e** As in **b-c** for cursor movement onset alignment and the interval t ​= ​[-0.3, 0.3] s. Significant differences are highlighted (∗).

The intercept term corresponds to the grand average activity across all trials. For the start of trial alignment, it is equivalent to the average of Fig. 3c and d. We observed significant group level activations in occipital, parieto-occipital and sensorimotor areas (Fig. 4a, top row). Interpreting the intercept term is not straightforward. If there is no condition- or direction-related activity, the intercept indicates activity that is common across all trials. At t ​= ​3.1 ​s, for example, the significant intercept term for voxels in parieto-occipital areas was due to similar activation in both conditions (Fig. 3c and d). If there are condition and/or direction-related effects, the intercept term represents the average. For example, at t ​= ​0.0 ​s in the case of cursor movement onset alignment, the intercept (Fig. 4b, top row) and condition (Fig. 4b, middle row) terms of voxels in medial central sensorimotor areas were significant. In this case, the intercept does not reflect common neural activity; it is simply the average of both conditions (Fig. 3g and h).

For the start of trial aligned condition factor (Fig. 4a, middle row), we observed significant voxel activity in contralateral sensorimotor areas from 2.3 ​s (300 ​ms after the condition cue) to 2.7 ​s, and from 3.0 ​s to 3.6 ​s. In the interval 3.1 ​s–3.4 ​s the activity intensified in central sensorimotor areas with a peak close to the average cursor movement onset (t ​= ​3.2 ​s). We additionally observed significant activity in occipital and parietal areas in the interval 2.0 ​s–2.8 ​s. For the cursor movement onset alignment (Fig. 4b, middle row), we also observed condition related activity in sensorimotor areas. The activity peaked in central sensorimotor areas at the cursor movement onset (t ​= ​0.0 ​s). We did not observe significant activity in occipital and parietal areas. Comparing the sensorimotor activity between the start of trial (Fig. 4a, middle row, t ​= ​3.2 ​s) and cursor movement onset (Fig. 4b, middle row, t ​= ​0.0 ​s) alignments, we observed a stronger effect of condition in the cursor movement onset alignment. This suggests that the sensorimotor activity, generating the hand MRCP, had a stronger phase-locking to the cursor movement onset.

For the start of trial aligned direction factor (Fig. 4a, bottom row), we observed significant voxel activity in parieto-occipital areas from 2.8 ​s to 2.9 ​s, corresponding to 300 ​ms–400 ​ms after the direction cue and 100 ​ms–200 ​ms after the average eye movement onset. The parieto-occipital cortex was again significantly activated from 3.2 ​s to 3.3 ​s, corresponding to 0 ​ms–100 ​ms after the average movement onset and 300 ​ms–400 ​ms after the target stimulus reached its final location. For the cursor movement onset aligned direction factor (Fig. 4b, bottom row), we did not observe significant activity. The lack of significant activity in the cursor movement onset alignment indicates that the parieto-occipital activity was phase-locked to the start of trial alignment.

### 3.4 Differences in direction encoding

We also investigated if the encoding of directional information varied between conditions. We observed a tendency that areas along the dorsal stream encoded more information around the cursor movement onset in the execution condition (Fig. 5b,d). For the start of trial alignment (Fig. 5b and c), we observed a weak effect in PM of both hemispheres (t_left_ ​= ​3.3, t_right_ ​= ​4.6); the effect did not turn significant at the ROI level (Fig. 5c). For the cursor movement onset alignment, all ROIs tended to encode slightly more directional information (Fig. 5e). The *t*-test results were not significant (except for PO-right) because of considerable variance across participants compared to the mean difference. This suggests that EEG delta band activity that originated in sensorimotor areas tended to encode more directional information in the execution condition. The size of the effect (mean difference between conditions ​≤ ​0.7) for the activity in sensorimotor areas was small and less consistent across participants compared to the parieto-occipital activity present in both conditions (Fig. 4a, bottom).

### 3.5 Single time-point condition and direction classification

**Fig. 6:**
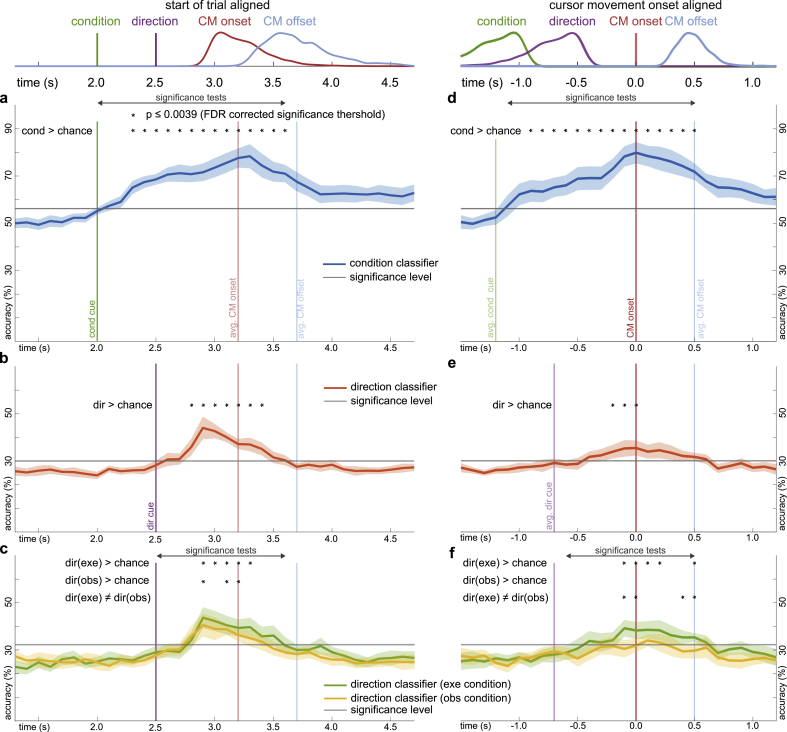
Grand average condition and direction classification accuracy curves. For each time-point, an sLDA classifier was fit in a cross-validation scheme to predict condition or direction from the EEG channels. All plots show the grand average test set accuracies and their confidence intervals across participants. **a,** Condition (exe vs. obs) classification accuracy curve for start of trial aligned data. To identify significant group level effects, we computed one-sided permutation paired t-tests (critical p value ​= ​0.0039, FDR corrected) between the test set accuracies (blue) and participant specific significance levels (gray). Significant differences are highlighted (∗). **b,** As in **a** for direction (right vs. up vs. left vs. down) classification accuracy curves. **c,** Condition specific direction classification accuracy curves. In addition to one-sided tests against the significance level, we computed two-sided permutation paired t-tests between the exe and obs specific accuracy curves to identify significant interaction effects between condition and direction. **d-f,** As in **a-c** for cursor movement onset aligned data.

Fig. 6 shows accuracy curves (condition, direction) for single time-point classifiers and both alignment methods. We compared accuracy curves with the significance levels to identify group level effects (Fig. 6a–c,d-f), while we identified interaction effects (condition ​× ​direction) by comparing direction classifier accuracy curves across conditions (Fig. 6c,f).

The condition classifier accuracy curves for start of trial (Fig. 6a) and cursor movement onset aligned data (Fig. 6d) crossed the significance level threshold of 56.0% (0.6% SD) following the condition cue. The start of trial aligned accuracy reached its peak of 78.4% (9.2% SD) around the average cursor movement onset (t ​= ​3.3 ​s); the cursor movement onset aligned peak was 79.8% (8.2% SD) at t ​= ​0.0 ​s. In either alignment, the accuracy declined within 1 ​s after the peak and eventually plateaued. We observed significant group level accuracies from 2.3 ​s to 3.6 ​s for start of trial (Fig. 6a) and from −0.9 ​s to 0.5 ​s for cursor movement onset aligned data (Fig. 6d).

In Fig. 6b,e, we show accuracy curves for direction classifiers that were fit to distinguish between the four movement directions (right, up, left, down) irrespective of condition. We obtained significant group level effects for both alignments (Fig. 6b,e). The accuracies exceeded the significance level of 30.0% (0.46% SD) from 2.8 ​s to 3.4 ​s for start of trial (Fig. 6b), and from −0.2 ​s to 0.0 ​s for cursor movement onset aligned data (Fig. 6e). For each type of alignment, the accuracy peaked at 44.0% (8.6% SD) at 2.9 ​s and 35.5% (5.7% SD) at 0.0 ​s, respectively.

Lastly, we report direction classifier accuracy curves for each condition (Fig. 6c,f). We compared the group level averages with a significance level of 32.3% (0.8% SD) in execution and 32.0% (0.8% SD) in observation condition. For start of trial aligned data (Fig. 6c), the execution condition curve was above the significance level from 2.9 ​s to 3.3 ​s, while the observation condition curve was significant at 2.9 ​s, 3.1 ​s and 3.2 ​s. There was no significant difference between conditions (two-sided permutation paired t-tests, dir(exe) ≠ dir(obs)). That is, there was no interaction effect. For cursor movement onset aligned data (Fig. 6f), the curves in observation condition were not significant, while in execution condition we observed significant accuracies from −0.1 ​s to 0.2 ​s and at 0.5 ​s. There was also a significant difference between conditions at similar time-points (two-sided permutation paired t-tests, dir(exe) ≠ dir(obs)). These results suggest that direction could be inferred from the EEG delta band activity for start of trial aligned data irrespective of condition (Fig. 6c). Whereas for cursor movement onset aligned data, information about direction could be inferred only in execution condition (Fig. 6f).

### 3.6 Start of trial alignment vs. cursor movement onset alignment

**Fig. 7:**
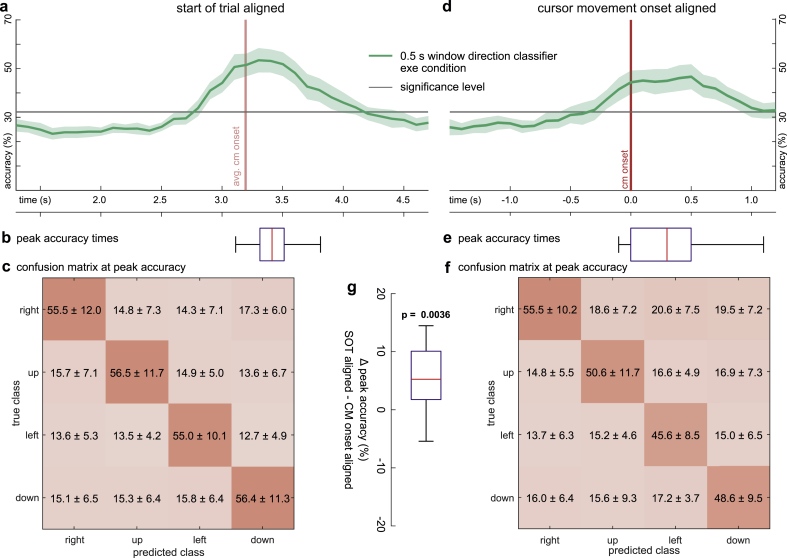
Execution condition. Effect of alignment on direction classification accuracy. For each time-point, an sLDA classifier was fit to predict the direction from EEG activity at the current and 5 preceding time-points (windowed classification). **a,** Windowed classification accuracy curve for start of trial aligned data. Shaded areas indicate the confidence interval across participants. **b,** Boxplot summarizing the participants’ peak accuracy time-points. **c,** Confusion matrix at the peak accuracy. **d-f,** As in **a-c** for cursor movement onset aligned data. **g,** Boxplot summarizing the paired difference in peak accuracy between the two alignments. The start of trial aligned peak accuracy was significantly higher than the cursor movement aligned one (p ​= ​0.0036, critical p-value ​= ​0.0039, FDR correction for 132 tests).

We used the windowed classification approach to investigate the effect of alignment on direction classification in execution condition. Fig. 7a,d shows the accuracy curves for both alignment types. The windowed classifiers achieved peak accuracies of 55.9% (8.6% SD) at 3.4 ​s (0.2 ​s SD) for the start of trial and 50.6% (7.5% SD) at 0.3 ​s (0.3 ​s SD) for the cursor movement onset aligned data. The confusion matrices for the peak accuracies indicate no bias towards one of the 4 directions for the start of trial alignment (Fig. 7f) and a small bias towards rightward movements for cursor movement onset alignment (Fig. 7f). We observed significantly higher peak accuracies for the start of trial alignment than for the cursor movement onset alignment (Fig. 6g, average Δ peak accuracy 5.3%, p ​= ​0.0036, two-sided, permutation, paired *t*-test). This result suggests that the representation of movement direction within delta band EEG activity was more consistent across trials if the data was aligned to the cues, rather than to the cursor (hand) movement onset. The same holds true for the observation condition (Supplementary Fig. 4).

## 4 Discussion

We have studied the relation of MRCPs and directional information in the EEG delta band activity during center-out reaching movements. Our results indicate that delta band amplitude modulations carry significantly more directional information phase-locked to the direction cue compared to the cursor movement onset. This suggests a functional dissociation between the network generating the MRCP in the execution condition (movement initiation) and the network processing directional information (movement preparation). In addition to the functional dissociation, we observed a spatial separation. The network generating the hand movement-related MRCP in the execution condition comprised contralateral and central sensorimotor areas, while the network processing directional information in both conditions comprised medial parieto-occipital areas.

In the start of trial alignment case, we could infer directional information in both conditions, suggesting an effector (hand, eye) independent representation that was consistently expressed in medial parieto-occipital cortex. We found significant direction related activation 300–400 ​ms after the direction cue and again 300–400 ​ms after the target reached its final location (Fig. 4a, bottom row). This is in agreement with the results obtained from the PTT (Kobler et al., 2018). Using a regression approach to decode directional kinematics (position and velocity) we found that the medial parieto-occipital areas encoded directional information in both conditions. The medial parieto-occipital cortex in humans is functionally equivalent to V6/V6A in non-human primate areas (Pitzalis et al., 2015). V6 (Pitzalis et al., 2006) and V6A (Tosoni et al., 2015) have a retinotopic organization in humans and are active in reaching and eye movements (Fabbri et al., 2010; Fernandez-Ruiz et al., 2007; Filimon et al., 2009; Magri et al., 2019). It is, therefore, unlikely that the direction related activity in the parieto-occipital cortex simply reflects visually evoked potentials.

For the cursor movement onset aligned data, significant directional information was present from −100 to 200 ​ms in the execution condition (Fig. 6f), suggesting a hand movement specific representation around the cursor movement onset. We did not observe a consistent group level representation of direction on the cortical surface during this interval (Fig. 4b, bottom row). If the voxels’ dipole orientations were allowed to vary across participants, we observed slightly higher direction related activity in sensorimotor areas in execution condition in contrast to observation condition (Fig. 5d and e). This suggests that hand movement specific directional information, originating in sensorimotor areas, was present in the delta band EEG signals and that its encoding varied across participants. Other studies reported significant SM1 activity during center-out movements (Bradberry et al., 2010; Waldert et al., 2008). If single or few joint movements are used to control the cursor movement, then muscle activity and direction are strongly correlated. Since, SM1 has a somatotopic organization, different joint movements are spatially separated and a classifier can extract this information from EEG activity (Ofner et al., 2017). Despite that there is clear evidence from invasive studies that directional information is present in SM1 (Ball et al., 2009; Georgopoulos et al., 1982; Mehring et al., 2003). However, the spatial distribution of preferred directions of SM1 neurons is approximately random (Amirikian and Georgopulos, 2000; Ben-Shaul et al., 2003). Hence, the probability of observing direction related effects at an electrode becomes lower as the number of neurons, contributing to the potential at the electrode, increases (Hammer et al., 2016). It is, therefore, unlikely to obtain a strong group-level effect of direction in the EEG activity generated in SM1.

Using the windowed approach to classify directions, we obtained peak accuracies of 55.9% (8.6% SD) for the start of trial alignment. Waldert et al. also investigated the classification of directions from EEG activity for executed hand movements in 4 directions (Waldert et al., 2008). They aligned the data to the movement onset and reported peak classification accuracies of 55.0% (SD 8.4%) 500 ​ms after the movement onset. Wang and Makeig used a similar paradigm to investigate movement direction in a pilot study (4 participants). They reported that EEG activity originating in PPC could predict direction (left vs. right) 200–300 ​ms after the direction cue with an accuracy of 80% irrespective of hand, eye or hand ​+ ​eye movements (Wang and Makeig, 2009). Later (Li et al., 2012), reported an accuracy of 65.4% for the same task with a larger sample (10 participants). Wang et al. also studied a visuomotor center-out task using wrist movements (Wang et al., 2010). They predicted 4 directions from delta band MEG signals, and reported higher classification accuracies if the participants could move immediately after target presentation rather than a forced random delay period. This is in agreement with our finding that significantly higher classification accuracies could be obtained cue aligned compared to movement onset aligned (Fig. 7, Supplementary Fig. 4).

The strongest difference in EEG activity between the two experimental conditions can be attributed to the MRCP related to the hand movement initiation (Fig. 3). The MRCP waveform (Fig. 3f) and cortical activation in central sensorimotor areas (Fig. 3g) in the execution condition agree with previous EEG studies which investigated upper-limb movements (Jochumsen et al., 2013; Ofner et al., 2017). In the observation condition, we observed a slight negativity at electrode C1 (Fig. 3b,f), which was more pronounced for the start of trial alignment (0.7 ​μV) than for the cursor movement onset alignment (0.2 ​μV). The source space activity (Fig. 3d,h) indicates that central sensorimotor areas were also activated in the observation condition, but to a significantly weaker extent than in the execution condition (see condition factor encoding in Fig. 4a and b). The single-lag condition classifiers utilized this difference and reached the highest classification accuracies at the movement onset (Fig. 6a,d). We did not observe a difference in the peak accuracies between the two alignment methods.

In the classification analysis, we observed an interaction effect between the factors condition, direction and alignment. The direction classifiers aligned to the cursor movement onset in the execution condition had significantly higher accuracies around the cursor movement onset than in the observation condition (Fig. 6f). Note that the cursor was not controlled by the participant in the observation condition and, therefore, not task relevant. This could explain why the classification accuracies were at chance level in observation condition (Fig. 6f, yellow curve). The execution condition classification accuracies were significantly higher than chance level, indicating the presence of directional information around the movement onset (Fig. 6f, green curve). As pointed out above, we did not observe a consistent group level encoding of direction in the cortex for the cursor movement alignment; however, we observed a tendency that areas along the dorsal stream encoded more information in the execution condition (Fig. 5d and e). The direction classifiers utilized this participant, execution condition and cursor movement onset specific representations to achieve significant accuracies (Fig. 6f).

Since the participants were moving their eyes and right arm, artifacts could have contaminated the EEG signals. In the EEG delta band corneo-retinal dipole and blink artifacts are the dominating eye artifacts (Keren et al., 2010). Using a state of the art correction algorithm (Kobler et al., 2020b) we could remove their contribution. Supplementary Figs. 7 and 8 show the encoding results without eye artifact correction. As expected, we observed strong direction-related activity at cortical sites closest to the eyes (e.g., prefrontal and anterior temporal areas). After correction, we did not find such artifactual contributions (Fig. 4a and b; Supplementary Figs. 5 and 6). Hence, it is unlikely that eye artifacts contributed to the direction classification results. Regarding movement artifacts, the activity of the shoulder/neck muscles at the start and stop of the arm movement could have contaminated the EEG during the execution condition. Indeed, activity in inferior temporal sites of the right hemisphere before the cursor movement onset and at the cursor movement offset (Fig. 4a and b; middle rows) could be explained by residual movement artifacts. Alternatively, the activity at these sites could reflect the activity of subcortical brain structures (e.g., cerebellum) that we did not include in our head model. It was recently demonstrated that EEG signals also contain brain activity originating in subcortical regions (Seeber et al., 2019). In either case, the activity was clearly weaker than the central sensorimotor cortex activity. Hence, we think that the hand movement initiation related MRCP mainly contributed to the condition classification.

Studying two experimental tasks could have been another potential limitation. The center-out task was succeeded by the PTT in half the trials. Since we gave the participants time to practice both tasks, and the instructions across tasks were similar, we think that the confounding effects on behavior and EEG activity were negligible. Lastly, despite using state of the art methods to detect the movement onsets from the cursor trajectories, it is not possible to detect the exact onset. This could have introduced jitter that might have attenuated effects phase-locked to the movement onset. To ensure that the jitter was minimal, we visually inspected the automatically detected onsets and fine-tuned the parameters.

In this study, we investigated delta band amplitude modulations during center-out movements. In addition to the MRCPs in the delta band, it is established that the EEG alpha and beta band power in sensorimotor areas change during movements (Pfurtscheller and Lopes Da Silva, 1999). In the last years, it has been shown that these power changes also covary with upper-limb movement rhythmicity (Seeber et al., 2016), Euclidean norm of acceleration (Marty et al., 2018) and direction (Kobler et al., 2020a; Korik et al., 2018). It is not clear whether the delta band amplitude modulations or alpha/beta band power modulations contain more directional information about executed movements. Korik et al. studied a center-out task and reported that movement direction could be decoded with moderate correlations (approx. 0.4) from alpha and beta band power features and with low correlations (approx. 0.15) from delta band amplitude features. In a circular arm movement task, we observed the contrary; movement direction was highly correlated (approx. 0.68) with delta band amplitude modulations, while the correlations with beta band power modulations were moderated (approx. 0.28). A key difference to (Kobler et al., 2020a) is that the participants moved their eyes in this study. Although we corrected for corneo-retinal dipole (CRD) and eyelid artifacts, the eye movements introduce another type of artifact – the saccadic spike potential (SP) (Keren et al., 2010). The SP reflects extraocular muscle activity, whose contribution starts at about 10 ​Hz and dominates the CRD and eyelid artifacts for frequencies above 20 ​Hz. The SP artifact also varies with eye movement direction (Keren et al., 2010), which potentially confounds direction decoding results from band-power features. Taken together, further research is required to disentangle the SP artifact and direction related band power changes.

## 5 Conclusions

Previous behavioral work gave indirect evidence that movement initiation and movement preparation have distinct cortical representations (Haith et al., 2016). We found direct evidence that EEG delta band amplitude modulations carry information about both arm movement initiation and directional processing, as an integral part of movement preparation, and that they are represented in two distinct cortical networks. Information about movement direction was primarily expressed in parieto-occipital area activity and was phase-locked to the direction cue. Whereas information about the arm movement initiation was reflected in the MRCP, a response phase-locked potential, originating in central sensorimotor areas. Despite generating the MRCP, the sensorimotor areas also showed a tendency to encode information about movement direction; compared to the parieto-occipital area activity, the direction-related activity in sensorimotor areas was less consistent across participants and specific to the execution condition. Using a classification approach, we demonstrated that significantly more information about movement direction can be inferred from the EEG activity for cue aligned data. This finding may prove useful in future non-invasive brain-computer interface designs.

## CRediT authorship contribution statement

**Reinmar J. Kobler:** Formal analysis, Writing - original draft, Writing - review & editing. **Elizaveta Kolesnichenko:** Formal analysis, Writing - original draft, Writing - review & editing. **Andreea I. Sburlea:** Writing - review & editing. **Gernot R. Müller-Putz:** Writing - review & editing.
