# Supplementary material for: Distinct cortical networks for hand movement initiation and directional processing: An EEG study
Source: Neuroimage. 2020 Oct 15;220:117076. doi: 10.1016/j.neuroimage.2020.117076 (PMC7573539; doi:10.1016/j.neuroimage.2020.117076)
Supplement: Multimedia component 2 [file mmc2.pdf]

Supplementary Material

Distinct cortical networks for hand movement initiation and directional processing: an EEG study

Reinmar J. Kobler[1,], Elizaveta Kolesnichenko[1,2,], Andreea I. Sburlea[1], Gernot R. Müller-Putz[1]*

[1] Institute of Neural Engineering; Graz University of Technology; Graz, Styria, 8010; Austria
[2] Swammerdam Institute for Life Sciences–Center for Neuroscience, University of Amsterdam; Amsterdam, North Holland, 1098XH; The Netherlands
These authors contributed equally.
* Correspondence: gernot.mueller@tugraz.at

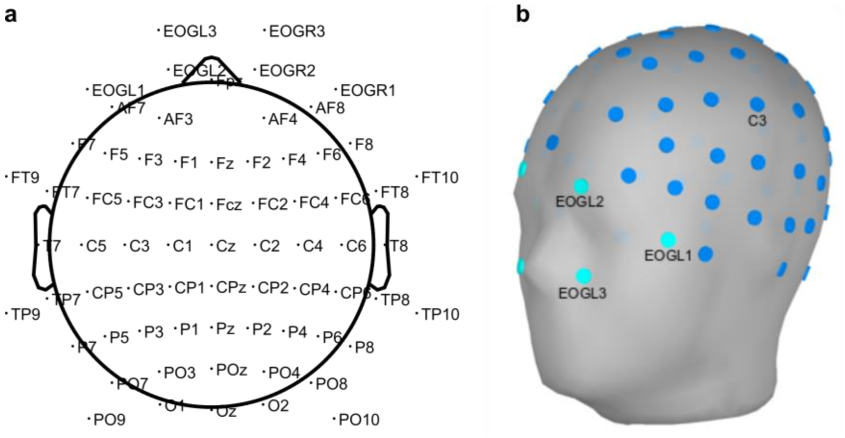

Supplementary Figure 1: High density EEG and EOG electrode locations. **a,** Topographical representation of EEG and EOG electrode locations. **b,** Grand average electrode locations on the template head model after co-registration with the recorded electrode positions.

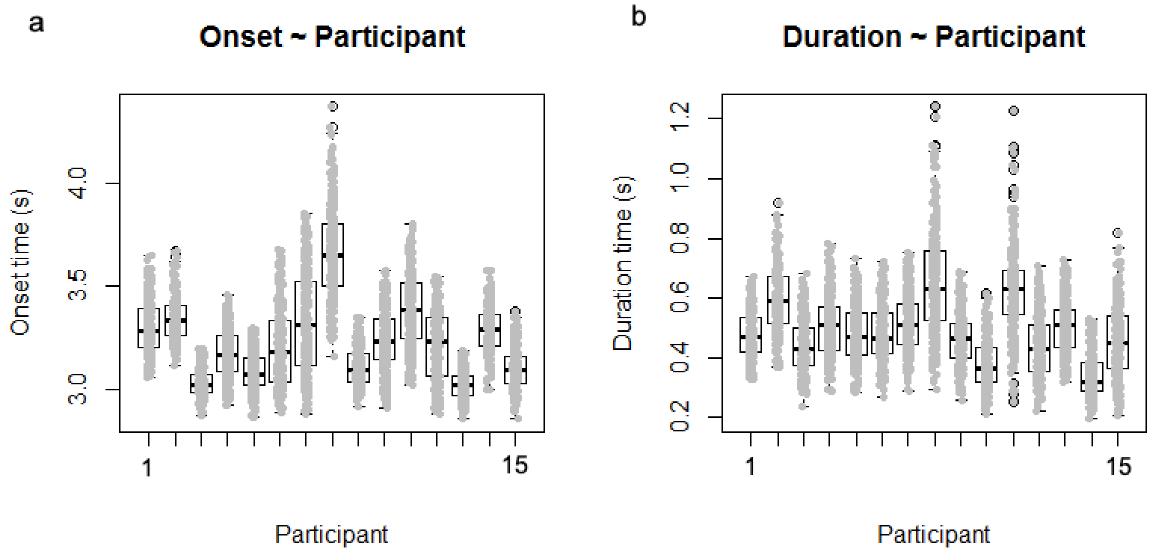

Supplementary Figure 2: Behavioral results per participant. **a,** Distribution of the detected cursor movement onsets across trials for each participant (n = 15). Each trial represents a gray dot. Boxplots summarize the distribution across trials. **b,** As in **a** for cursor movement duration.

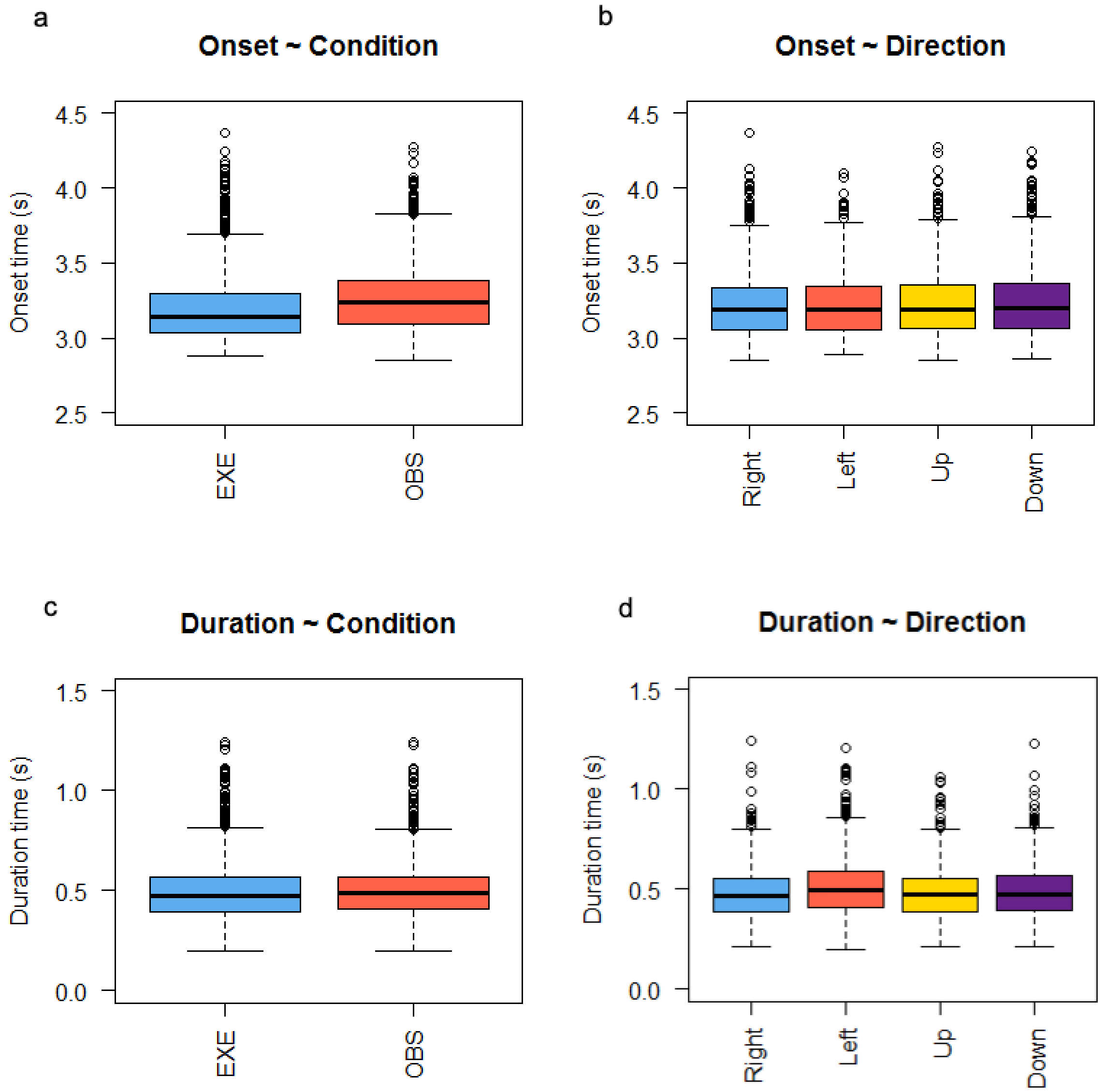

Supplementary Figure 3: Behavioural results for cursor movement onset and duration. **a,** Boxplots summarize the distribution of cursor movement onsets across trials and participants for execution condition (blue) and observation condition (red). **b,** As in **a** but grouped by direction instead of condition. **c-d,** As in **a-b** for the cursor movement duration.

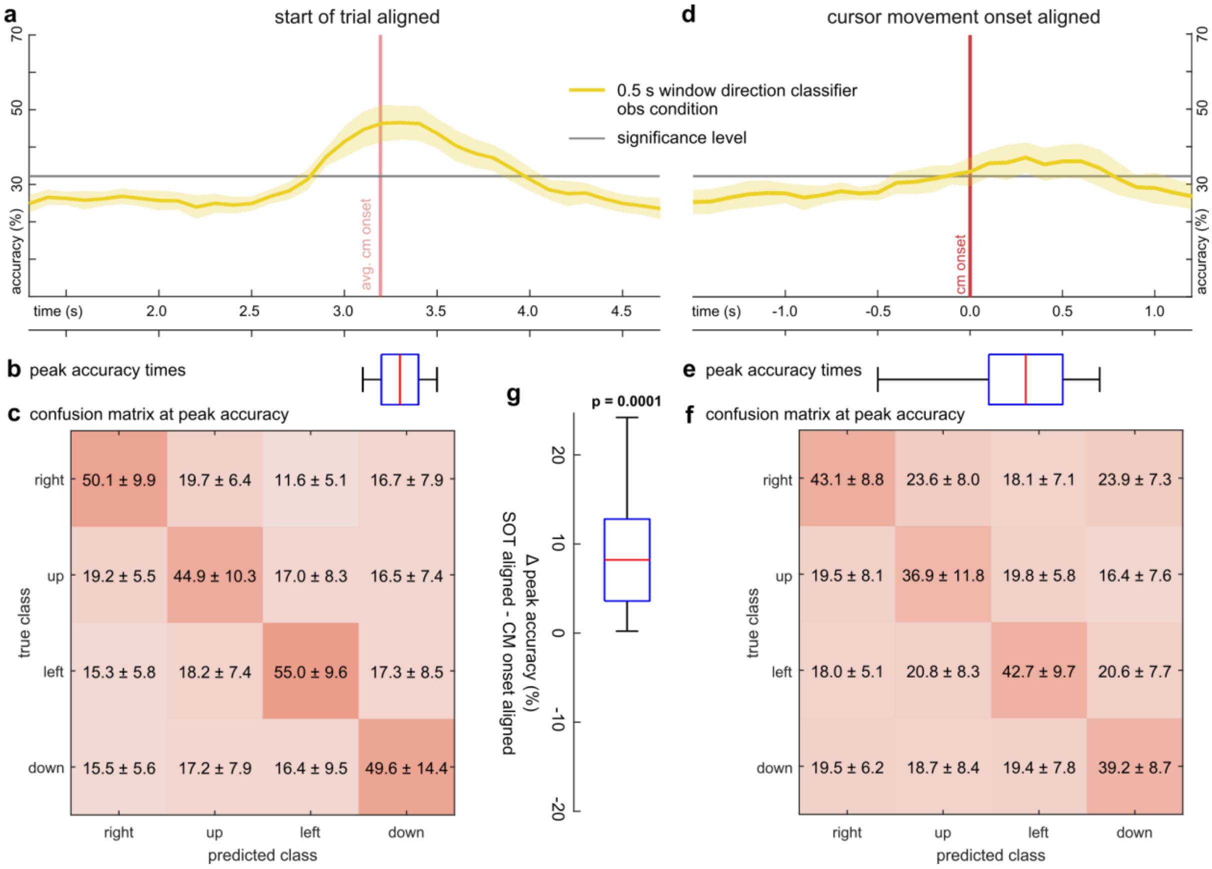

Supplementary Figure 4: Observation condition. Effect of alignment direction classification accuracy. For each time-point, a sLDA classifier was fit to predict direction from EEG activity at the current and 5 preceding time-points (=windowed classification). **a,** Windowed classification accuracy curve for start of trial aligned data. Shaded areas indicate the confidence interval across participants. **b,** Boxplot summarizing the participants' peak accuracy time-points. **c,** Confusion matrix at the peak accuracy. **d-f,** As in **a-c** for cursor movement onset aligned data. **g,** Boxplot summarizing the paired difference in peak accuracy between the two alignments. The start of trial aligned peak accuracies were significantly higher than the cursor movement aligned ones ($p = 0.0001$, critical p value = 0.0039, FDR correction for 132 tests).

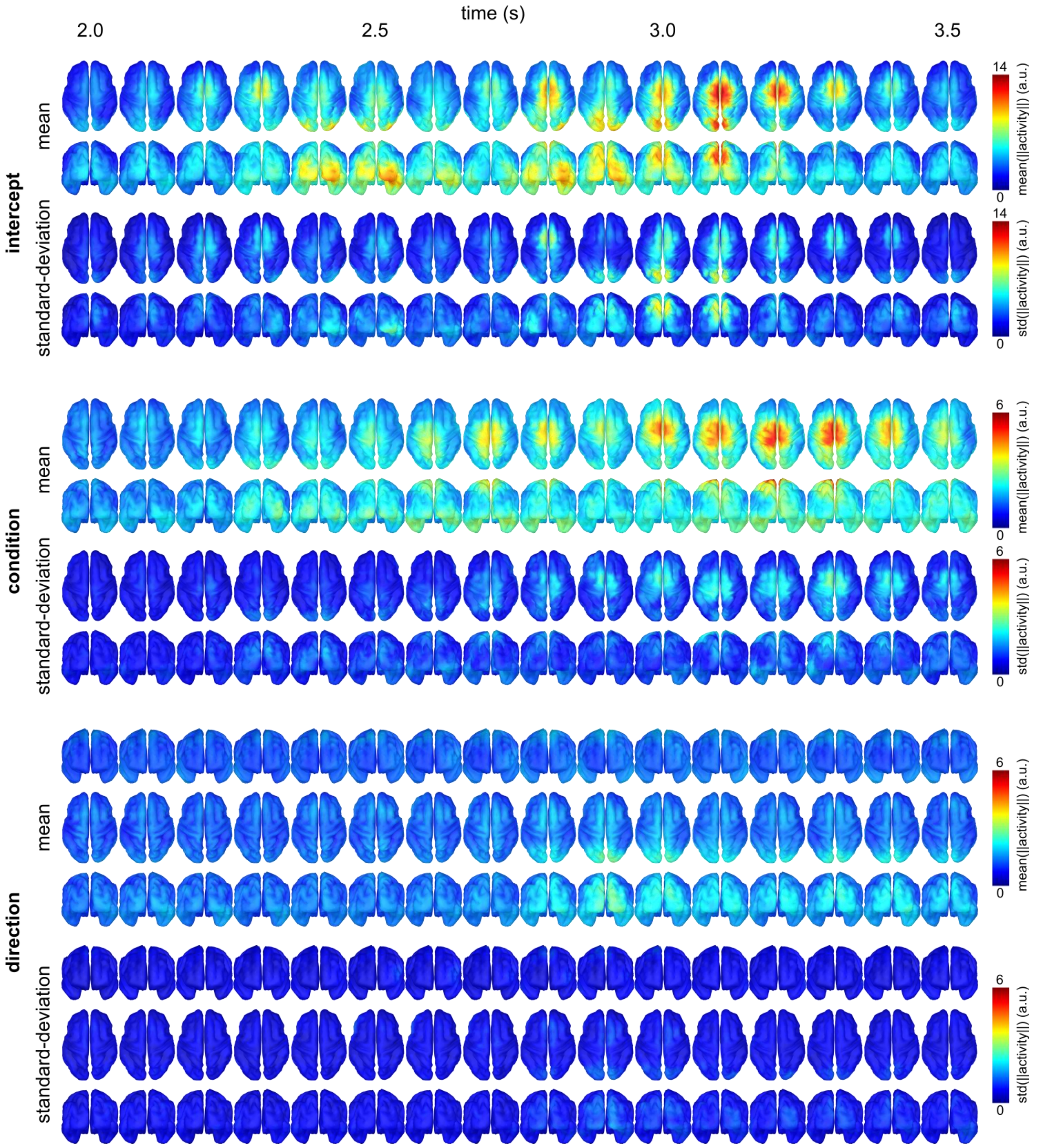

Supplementary Figure 5: Start of trial alignment. Group level source space encoding results. Mean and standard deviation across participants (n = 15) of the voxel norms for the intercept (top), condition (middle) and direction (bottom) factors. The horizontal and vertical direction factors were combined into a single direction factor by averaging the voxel norms. For each time-point, factor and summary statistic (mean, standard-deviation), the cortical activity is visualized with three views (front, top, back).

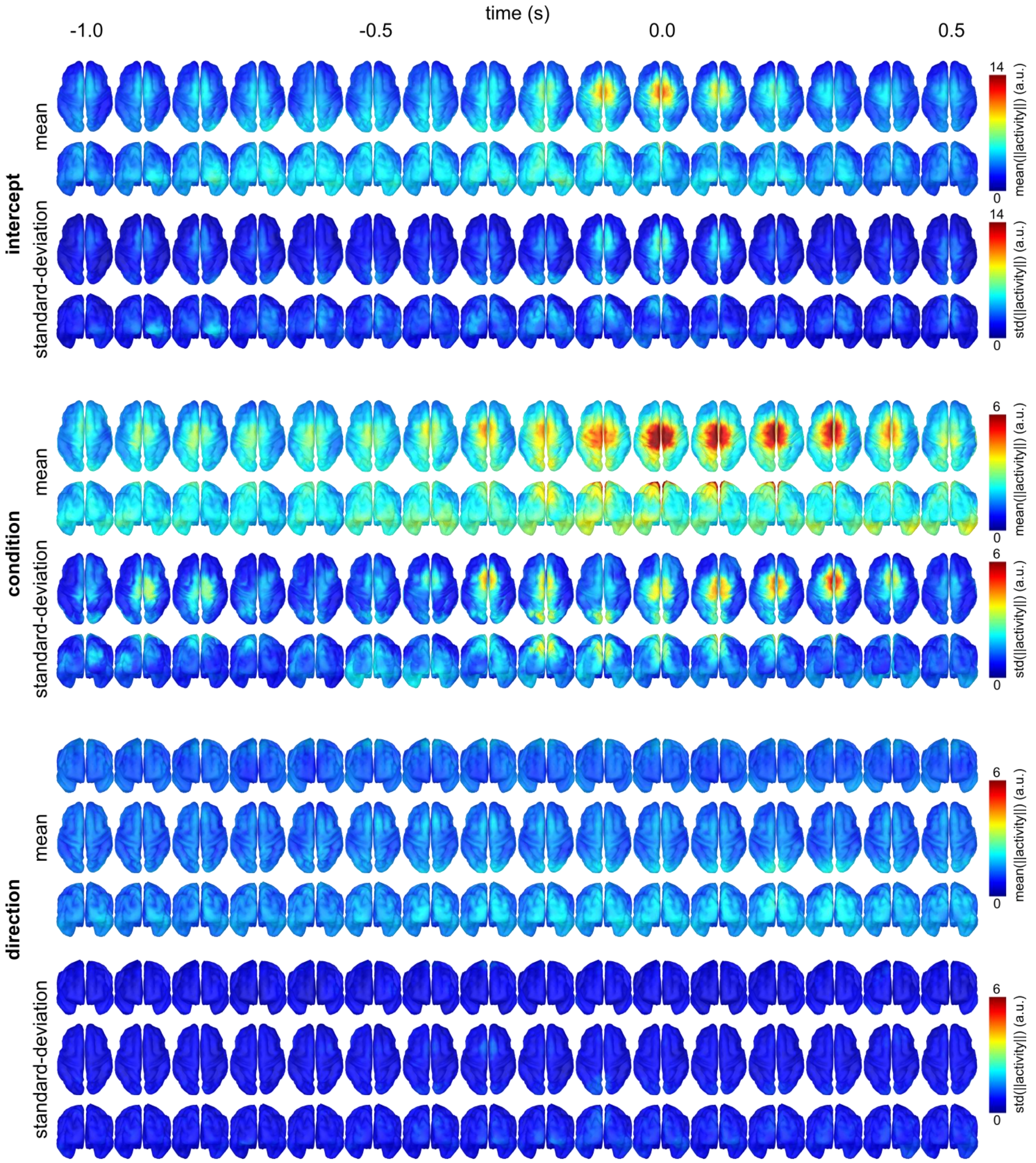

Supplementary Figure 6: Cursor movement onset alignment. Group level source space encoding results. Mean and standard deviation across participants (n = 15) of the voxel norms for the intercept (top), condition (middle) and direction (bottom) factors. The horizontal and vertical direction factors were combined into a single direction factor by averaging the voxel norms.

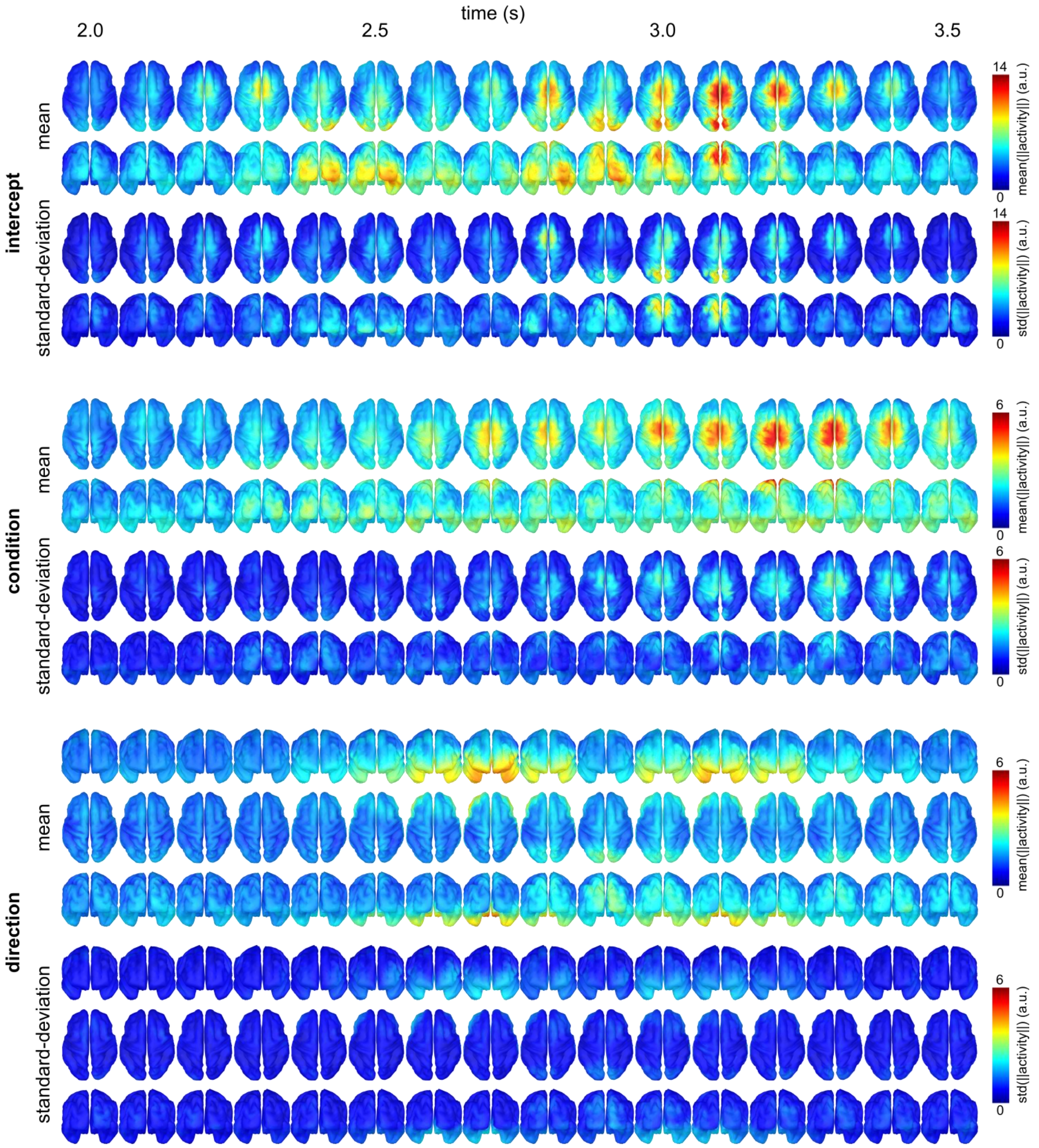

Supplementary Figure 7: Start of trial alignment. Group level source space encoding results without eye artifact correction. Mean and standard deviation across participants (n = 15) of the voxel norms for the intercept (top), condition (middle) and direction (bottom) factors. The horizontal and vertical direction factors were combined into a single direction factor by averaging the voxel norms.

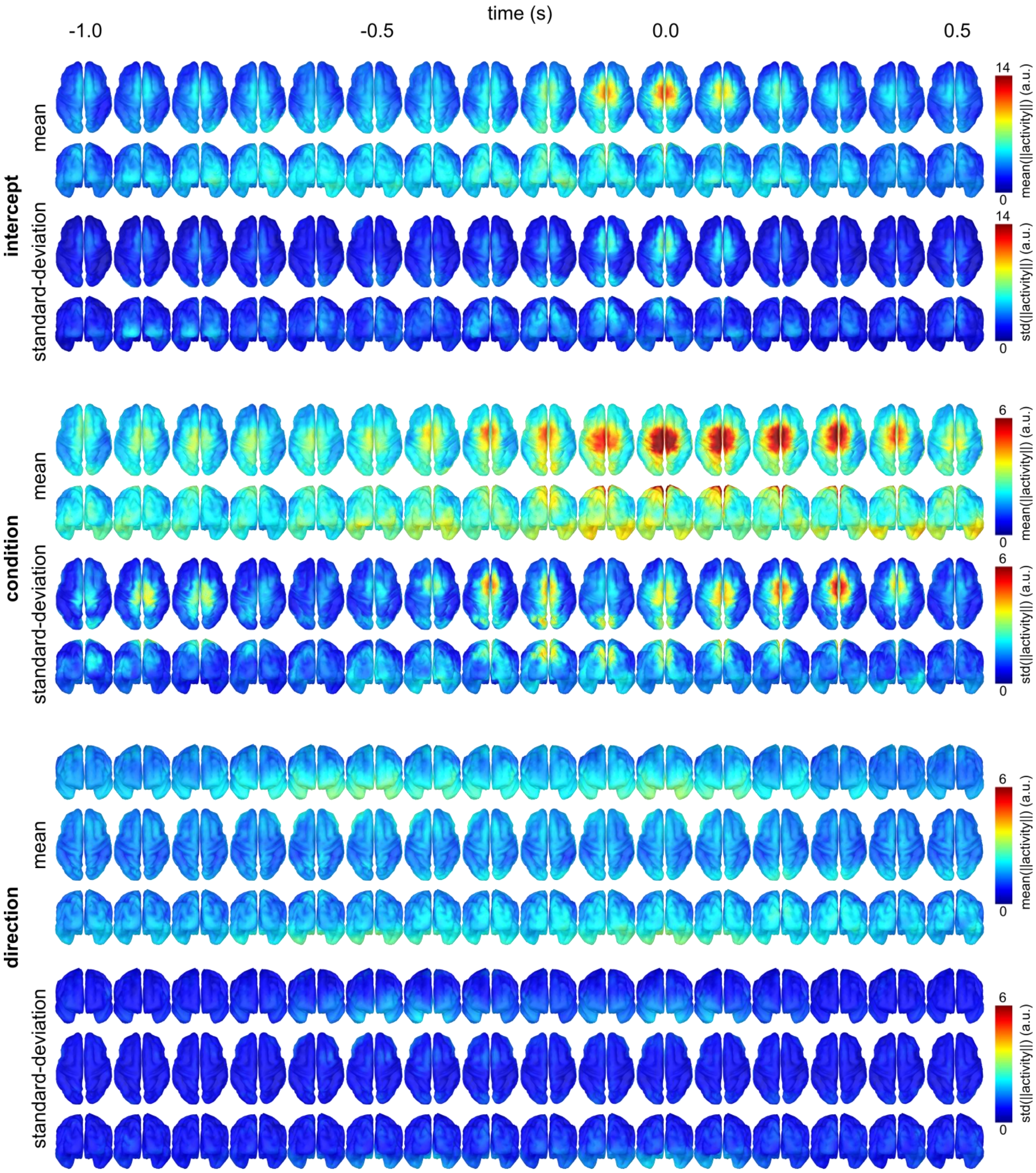

Supplementary Figure 8: Cursor movement onset alignment. Group level source space encoding results without eye artifact correction. Mean and standard deviation across participants (n = 15) of the voxel norms for the intercept (top), condition (middle) and direction (bottom) factors. The horizontal and vertical direction factors were combined into a single direction factor by averaging the voxel norms.

Supplementary Table 1: Data rejection summary. Column two indicates the interpolated EEG channels. Columns four, five and six list the number of trials marked for rejection by the application of three criteria: (1) EEG signal outliers (larger than $\pm$200μV, abnormal variance, probability or kurtosis); (2) trials with abnormal reach onset and (3) duration. The last column summarizes the number of trials rejected by combining all three criteria.

participant	interpolated channels	number of trials	outliers EEG	outliers reach onset	outliers reach duration	**total**
1	T7, T8, TP9	192	9	9	6	**20 (10%)**
2	T8, FT10, AF8	360	12	38	39	**67 (19%)**
3	FT10	362	2	14	15	**21 (6%)**
4	F5	361	14	32	37	**58 (16%)**
5	TP10, PO3	361	14	24	25	**41 (11%)**
6	TP9	363	22	24	21	**49 (14%)**
7	-	364	25	36	39	**65 (18%)**
8	TP7, TP8, TP9, TP10	361	45	33	34	**80 (22%)**
9	T7	360	20	34	28	**56 (16%)**
10	F7, AF8, T8, PO10	361	13	16	23	**40 (11%)**
11	TP10	364	55	34	33	**90 (25%)**
12	FC5, T8	363	28	22	21	**55 (15%)**
13	T8	360	20	23	29	**55 (15%)**
14	T7, T8, TP7, TP9, TP10	362	25	18	22	**54 (15%)**
15	FC3, TF7	360	24	15	14	**41 (11%)**
**average**	**2.1**	**350**	**22 (6%)**	**25 (7%)**	**26 (7%)**	**53 (15%)**
